# CZTS*_x_*Se_1−*x*_ nanocrystals: Composition dependent method of preparation, morphological characterization and cyclic voltammetry data analysis

**DOI:** 10.1016/j.dib.2016.07.026

**Published:** 2016-07-19

**Authors:** Yogesh A. Jadhav, Pragati R. Thakur, Santosh K. Haram

**Affiliations:** Department of Chemistry, Savitribai Phule Pune University (Formerly University of Pune), GaneshKhind, Pune 411007, India

**Keywords:** Synthesis, Characterization, CZTS*_x_*Se_1−*x*_ nanocrystals, Band edge parameters

## Abstract

In this article, synthesis procedures of preparation of copper zinc tin sulpho-selenide (CZTS*_x_*Se_1−*x*_) alloy nanocrystals and the data acquired for the material characterization are presented. This data article is related to the research article doi: http://dx.doi.org/10.1016/j.solmat.2016.06.030 (Jadhav et al., 2016) [1]. FTIR data have been presented which helped in confirmation of adsorption of oleylamine on CZTS*_x_*Se_1−*x*_. Transmission electron microscopy (TEM), Field emission scanning electron microscopy (FESEM) and atomic force microscopy (AFM) data have been presented which have been used to reveal the morphological details of the nanocrystals. The Energy dispersive X-ray analysis (EDAX) based elemental mapping data has been presented to confirm the elemental composition of nanocrystals. Procedure for the preparation of CZTS*_x_*Se_1−*x*_ based working electrode for the CV measurements have been given. The summary table for the optical, electrochemical band gaps, valance and conduction band edges as a function of composition are listed for the ready reference.

## Specification Table

**Table:** 

Subject area	*Chemistry*
More specific subject area	*Materials Chemistry/Electrochemistry*
Type of data	*Table, figure, images*
How data was acquired	*Instruments used- Bruker FTIR-ATR, Tensor-37 Spectrometer with a diamond ATR attachment, FESEM- FEI make Nova NanoSEM 450 FESEM with EDAX using Bruker XFlash 6I30. TEM-Philips CM200 TEM 200* *kV, AFM- Bruker, Multimode 8.0.*
Data format	*Analyzed*
Experimental factors	*CZTS*_*x*_*Se*_*1−x*_*(x=0–1) Nanocrystals with varied composition have been synthesized by hot injection method. These were characterized by FTIR, TEM, FESEM, AFM and EDAX analysis. Band edge parameters have been estimated by cyclic voltammetry.*
Experimental features	*The modified electrodes for the voltammetry measurements have been prepared by drop-casting the dispersion of samples. Cyclic Voltammetry measurements have been carried out in non-aqueous solvents under inert atmosphere.*
Data source location	*Savitribai Phule Pune University*, *Pune, Maharashtra, India*
Data accessibility	*Data is supplied with in this article*

## Value of the data

• Detail Solvothermal procedure for the preparation CZTS*_x_*Se_1−*x*_ (*x*=0–1) nanocrystals have been presented.
• Detail FTIR data analysis has been presented for the adsorbed oleylamine molecules on the CZTS*_x_*Se_1−*x*_ nanocrystals.
• AFM, TEM and FESEM data have been used to demonstrate composition independent morphological similarity of CZTS*_x_*Se_1−*x*_. The EDAX mapping data have been used to confirms the absence of phase segregation and formation of uniform composition.
• Composition dependent band edge positions have been presented in tabular form which will act as a guideline to design novel heterojunction photovoltaic device by band alignment engineering.

## 1 Data

The data given in this data article are in the form of seven figures and one table. It describes detail synthesis, characterization and procedure followed for cyclic voltammetry investigation of CZTS*_x_*Se_1−*x*_(*x*=0–1) nanocrystals. TEM data have been presented for highlight the morphology and size distribution. The topography has been recorded by FESEM and AFM image analysis. The EDAX spectra give the details about composition and stoichiometry. Uniform distributions of the constituent elements are seen in the elemental mapping images. The adsorption of capping agent, oleylamine has been confirmed by FTIR data. The procedure for the preparation of working electrode for the electrochemical investigation is described in detail.

## 2 Experimental design, materials and methods

### 2.1 Synthesis setup and CZTS*_x_*Se_1−*x*_ alloy nanocrystals synthesis

**Fig. 1:**
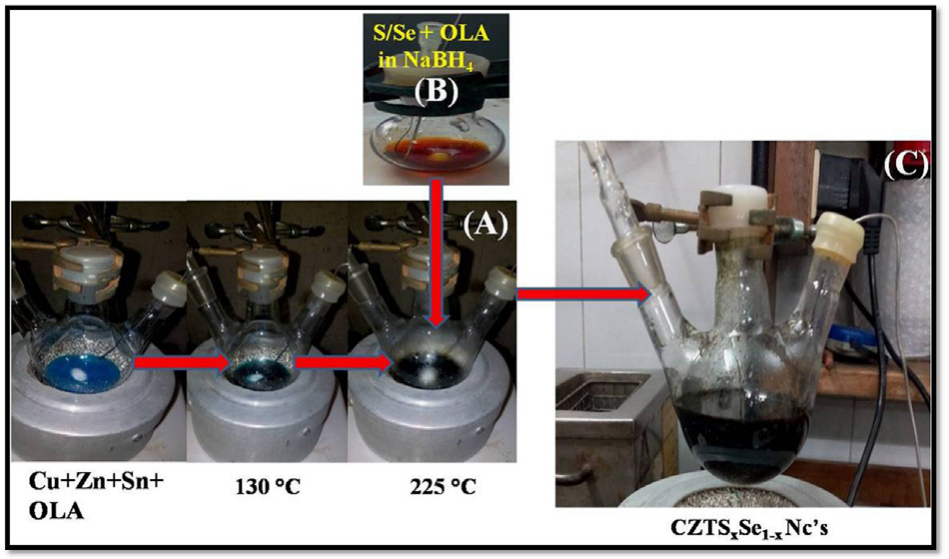
Experimental setup used and sequence of the addition followed in the synthesis of CZTS*_x_*Se_1−*x*_ (*x*=0–1) alloy nanocrystals.

The Synthesis of the CZTS*_x_*Se_1−*x*_(*x*=0–1) nanocrystals carried out using hot injection method suggested by Agrawal and Riha et al. [2], [3]. A typical synthesis setup used in present investigation [1] is shown in Fig. 1. The sequence of addition is marked as red-arrows. Weighed amount of all the constituents (salts in oleylamine (OLA)) are first transferred into the three necked flask, mounted on heating mantle, having temperature controller. Upon heating to 130 °C with constant stirring, all the metal complexes dissolved and from brown transparent solution. Temperature is raised further to 225 °C (Refer Fig. 1(A)) and into it freshly prepared S/Se in OLA solution was added, which leads to black color product (Refer Fig. 1(C)).

### 2.2 Drop-casting of films on the working electrode

The electrochemical measurements on the samples have been done on CZTS*_x_*Se_1−*x*_ coated gold-disk working electrode (2 mm diameter). For that, the sample was applied on the electrode by method of drop-casting. For that, the electrode was mounted vertically in a desiccator. 75 μL dispersion of nanocrystals in dichloromethane was applied carefully onto the electrode surface. The drop was allowed to dry by the help of mild vacuum (diaphragm vacuum pump) for 15 min. This helps solvents to evaporate quickly and form a film on the electrode surface. Adhesion of the film was found to be good and did not show any tendency to fall down or fouling in the solvent, during the measurements. From the weight of the loading, area of the electrode and density of samples, the thickness of the films were estimated and found to be in the range 58 μm. Such modified electrodes were used as prepared without any post annealing.

## 3 Characterization of CZTS*_x_*Se_1−*x*_ alloy nanocrystals

### 3.1 FTIR spectroscopy data on CZTS Sample

**Fig. 2:**
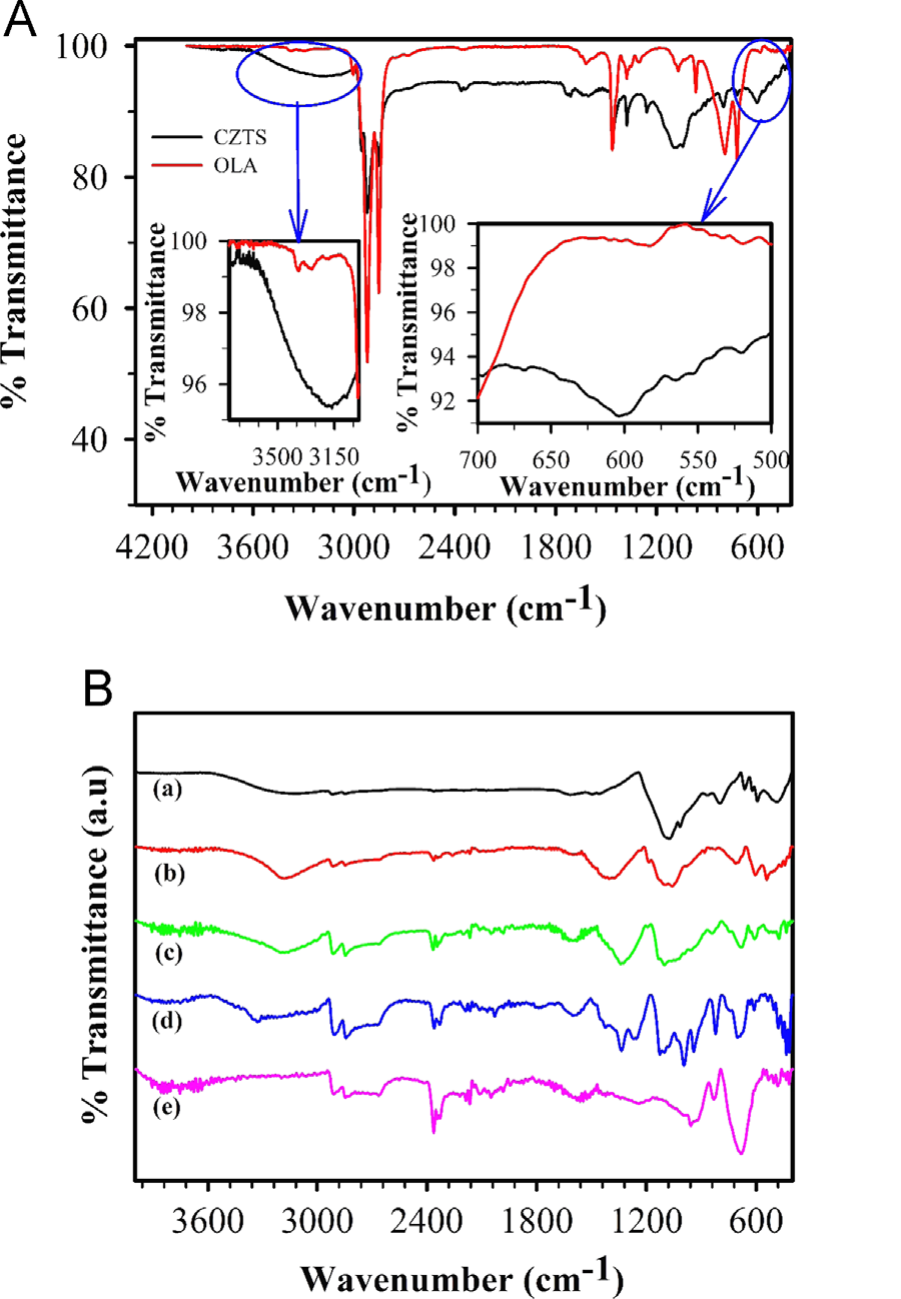
(A) FTIR spectra recorded on oleylamine (OLA) and CZTS nanocrystals. Inset shows the expanded region of the amine stretching and metals nitrogen bond characteristic stretching bands. (B) FTIR spectra for CZTS*_x_*Se_1−*x*_ nanocrystals with varied composition.

Fig. 2(A) shows the FTIR spectra recorded on CZTS nanocrystals powder samples and its comparison with a neat oleylamine sample (coordinating solvent). The band at 3372 cm^−1^ and 3292 cm^−1^ in OLA sample is attributed to the asymmetric and symmetric stretching from primary NH_2_ group [4]. These bands are replaced by new band at 3170 cm^−1^ in CZTS samples those matched with stretching vibration from the secondary amine group. This observation is explained on the basis of change in bond order during the adsorption. Similarly, the intensity of bending vibrations at 795 cm^−1^ for the NH_2_ in OLA is decreased and shifted to 802 cm^−1^ in CZTS sample. Intensity of the CH_3_ and corresponding to C–C bending vibrations at 1465 cm^−1^ and 722 cm^−1^ are decreased in case of OLA adsorbed on CZTS. In both the cases, neat OLA and OLA on CZTS show the C–H symmetric and asymmetric stretching vibrations [4] because of the methylene and methyl group at 2923 cm^−1^ and 2852 cm^−1^. The bending vibration due to C–N at 1071 cm^−1^ is broadened in case of OLA with CZTS. In CZTS sample a strong band at 605 cm^−1^ is observed which corresponds to metal nitrogen stretching mode [5]. Over all, from the FTIR data analysis, co-ordination of OLA with CZTS nanocrystals is confirmed. Fig. 2(B) shows the similar bands in case of rest all the composition.

### 3.2 Morphological, topological analysis of CZTS*_x_*Se_1−*x*_ nanocrystals by TEM, AFM and FESEM

**Fig. 3:**
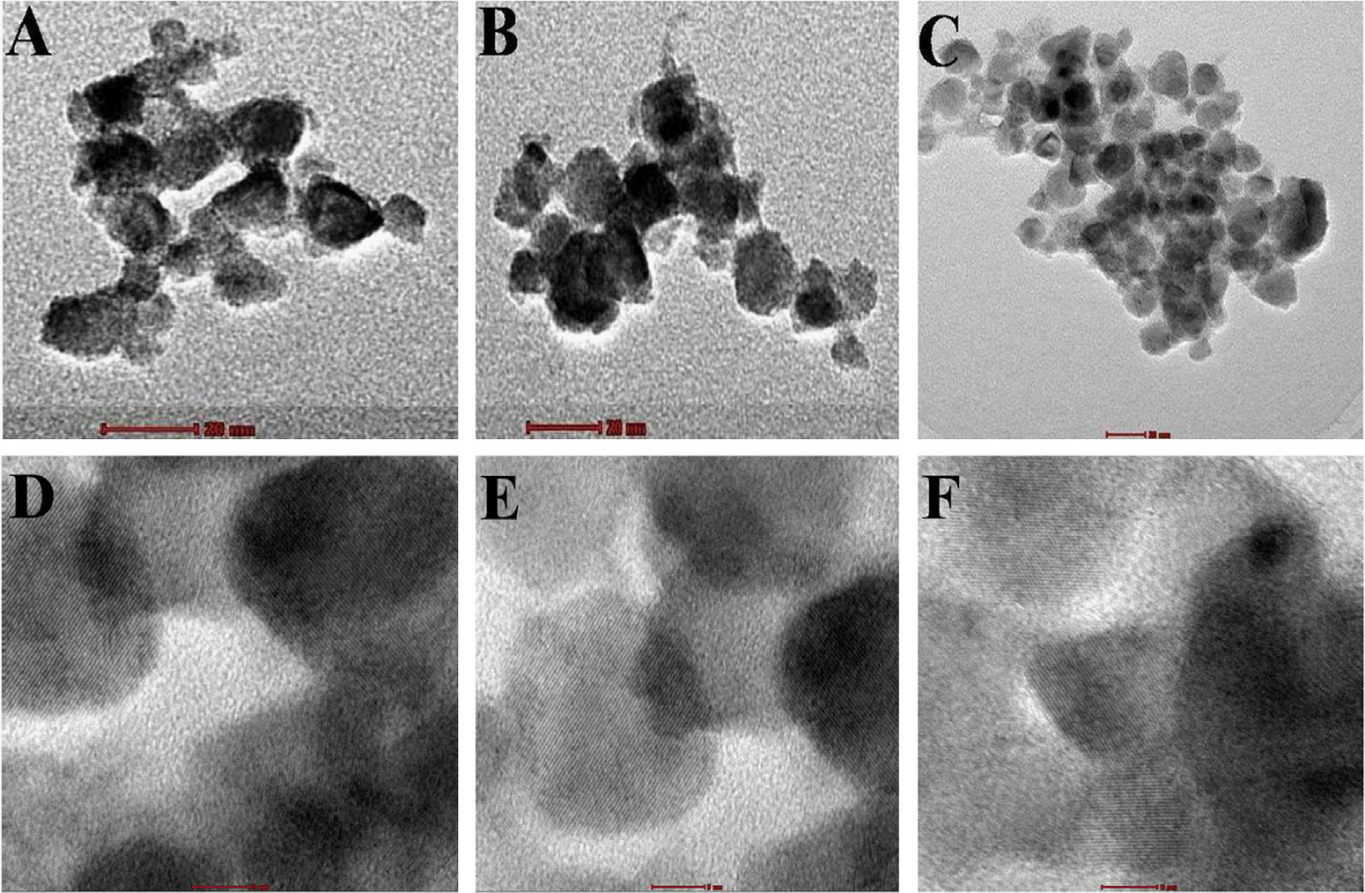
Low resolution TEM images recorded on CZTS*_x_*Se_1−*x*_ nanocrystals for varied composition. (A) x=0.17,(B) *x*=0.42, (C) *x*=0.74 and corresponding high resolution TEM images (D)–(F) respectively.

Fig. 3 shows the TEM micrographs for the CZTS*_x_*Se_1−*x*_ alloy nanocrystals for varied composition. Fig. 3(A) and (D) represents the low resolution and high resolution images for *x*=0.17 respectively, Fig. 3(B) and (E) represent the low and high resolution images for *x*=0.42 respectively, Fig. 3(C) and (F) represents the low and high resolution images for *x*=0.74 respectively. The TEM images for the *x*=1 and *x*=0 are shown in [1]. For all compositions faceted morphology with polyhedron shapes having size ranging from 10 to 30 nm are observed.

### 3.3 Stoichiometry/composition and elemental distribution of CZTS*_x_*Se_1−*x*_ nanocrystals by EDAX spectra and elemental mapping

**Fig. 4:**
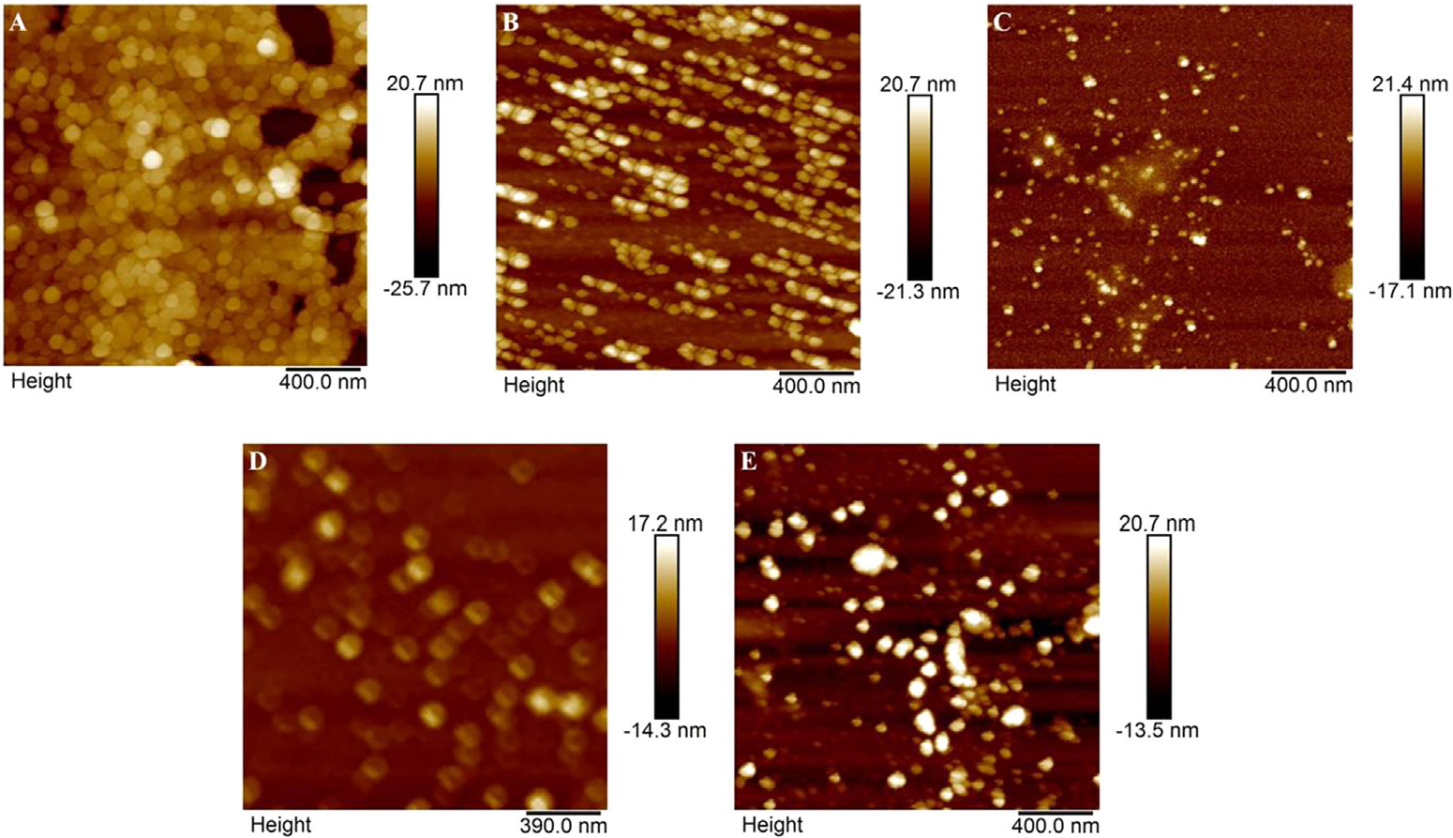
AFM images recorded on CZTS_x_Se_1-x_ nanocrystals dispersion dropcasted on silicon wafer for *x*=0–1. (A) *x*=1 (B) *x*=0.74, (C) *x*=0.42, (D) *x*=0.17 and (E) *x*=0.

Fig. 4(A–E) shows the AFM images recorded on CZTS*_x_*Se_1−*x*_nanocrystals for *x*=0–1. (A) *x*=1 (B) *x*=0.74, (C) *x*=0.42, (D) *x*=0.17 and (E) *x*=0. In the AFM images, the faceted and nearly spherical nanocrystals are observed. The average height for all the CZTS*_x_*Se_1−*x*_ nanocrystals is in the range of 20 nm, suggesting the uniform topology. The size distributions are in the range of 20–30 nm.

**Fig. 5:**
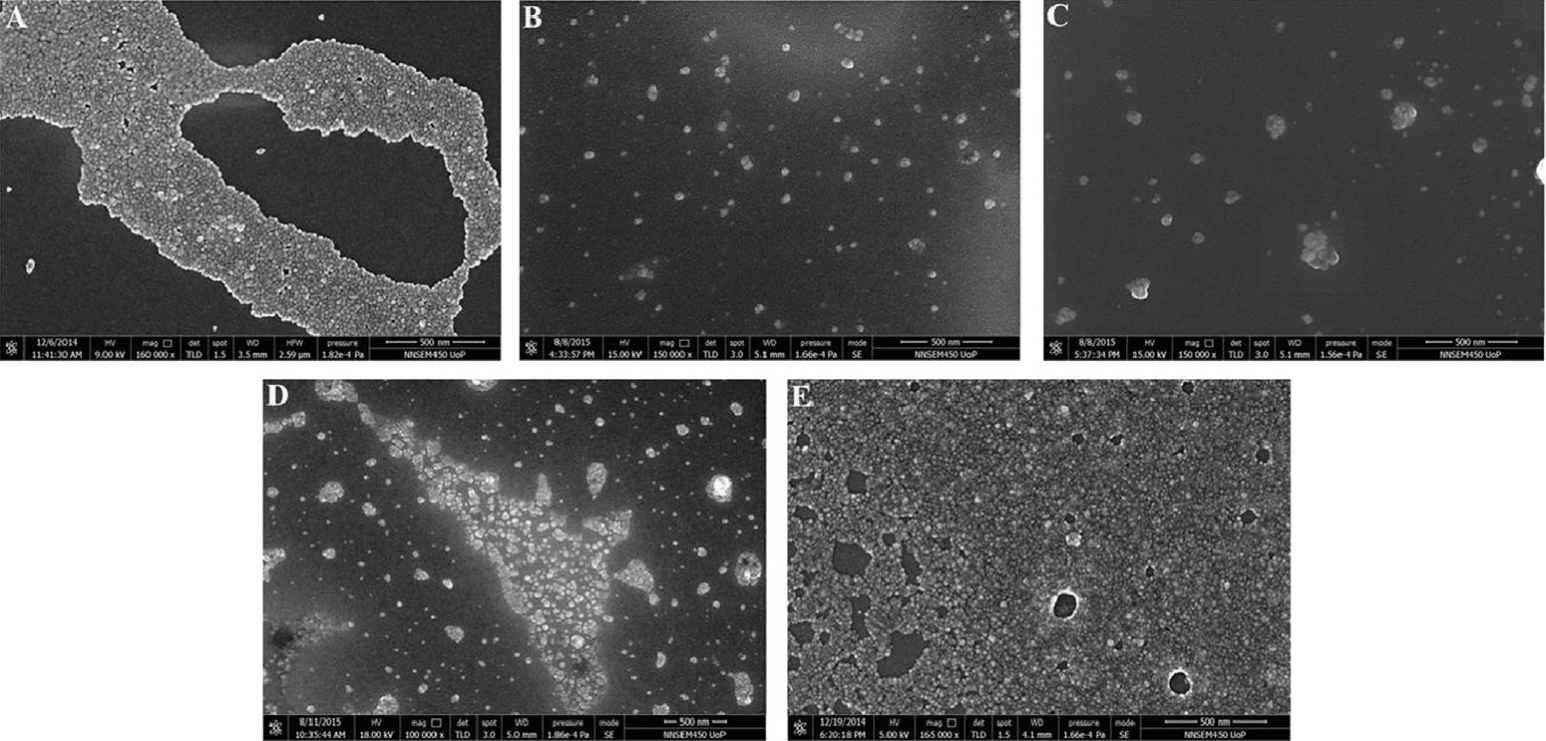
FESEM images recorded on CZTS*_x_*Se_1−*x*_ nanocrystals dispersion dropcasted on silicon wafer, for *x*=0–1. (A) *x*=1 (B) *x*=0.74, (C) *x*=0.42, (D) *x*=0.17 and (E) *x*=0.

Fig. 5(A–E) shows the FESEM micrographs recorded on CZTS*_x_*Se_1−*x*_ nanocrystals for *x*=0–1. (A) *x*=1 (B) *x*=0.74, (C) *x*=0.42, (D) *x*=0.17 and (E) *x*=0. The Fig. 5(A) and (E) show the mono-disperse nanocrystals spread over the silicon substrate. While Fig. 5(C)–(E) shows the poly disperse nature of nanocrystals. From the FESEM images, the size distributions were in the range of 10–30 nm and it matches very well with the size estimated from the TEM and AFM.

**Fig. 6:**
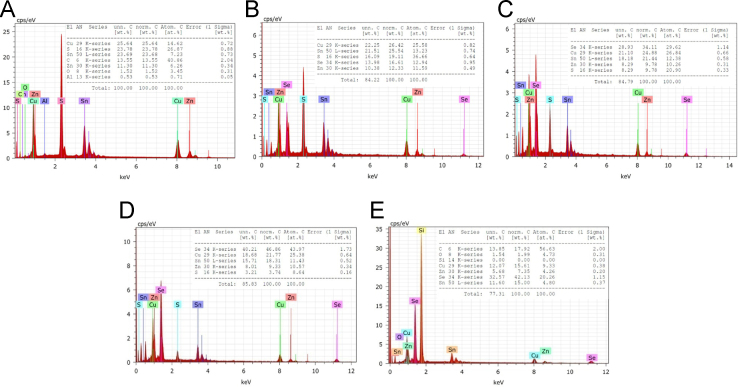
(A–E) EDAX Spectra recorded on CZTS*_x_*Se_1−*x*_nanocrystals for *x*=0–1. (A) *x*=1, (B) *x*=0.74, (C) *x*=0.42, (D) *x*=0.17 and (E) *x*=0. The inset shows the elemental distribution table obtained from EDAX.

**Fig. 7:**
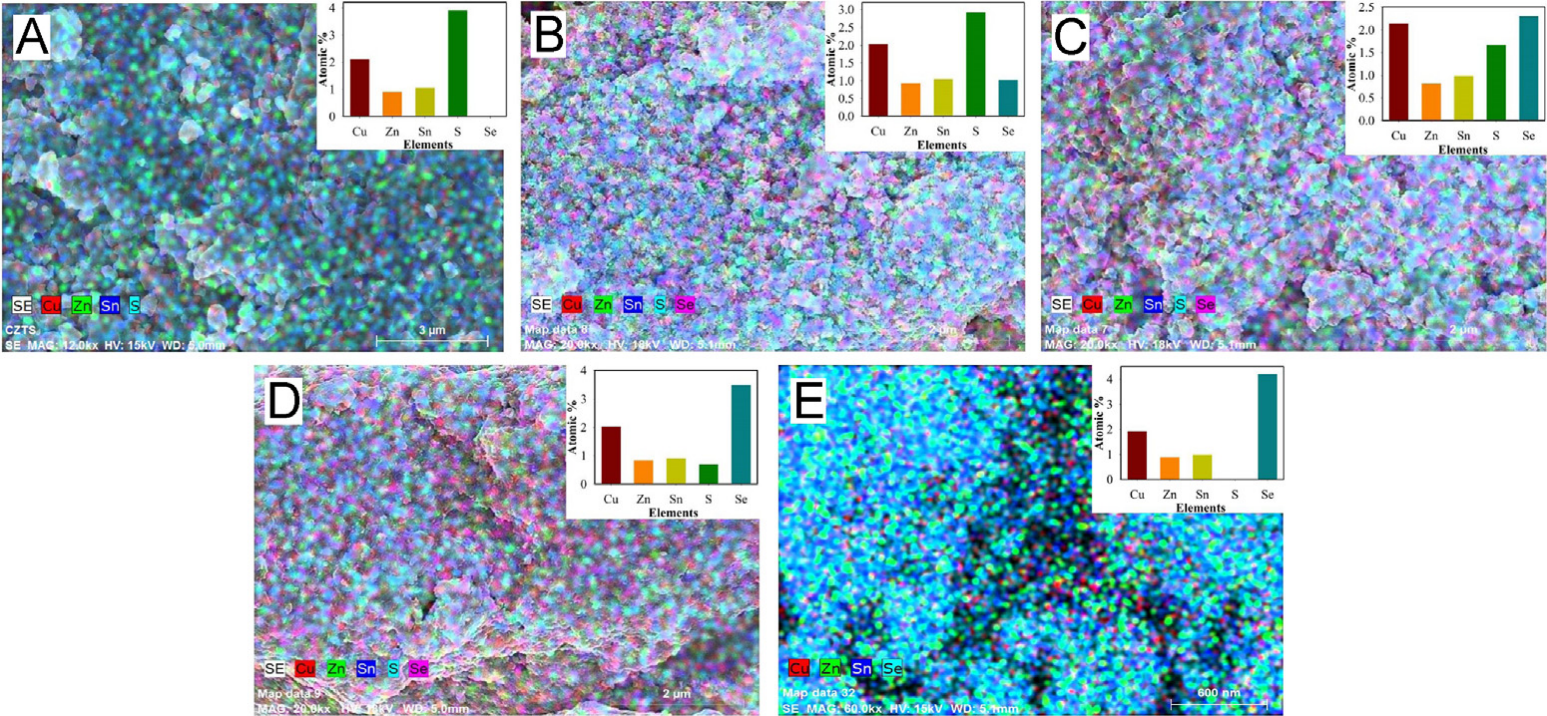
(A–E) shows the elemental mapping images for CZTS*_x_*Se_1−*x*_ alloys with ‘*x*’ ranging from 1 to 0. The insets show bar charts for stoichiometry.

Fig. 6(A–E) shows the EDAX spectrum recorded on CZTS*_x_*Se_1−*x*_ nanocrystals for *x*=0–1. (A) *x*=1 (B) *x*=0.74, (C) *x*=0.42, (D) x=0.17 and (E) *x*=0. From all the EDAX spectra, the compositions were estimated [1]. It shows good agreement between the ratios of precursor used in the synthesis and compositions estimated from EDAX. The distribution of elements in all the samples are measured by EDAX based elemental mapping. It is presented in Fig. 7(A–E). The inset of Fig. 7(A–E) shows the bar chart for stoichiometry as element vs. atomic % (stoichiometry). Mapping data underlines uniform stoichiometric distribution in the samples.

Table 1 summarizes the data for optical band gaps form UV–Visible spectroscopy and electrochemical band gaps from cyclic voltammetry. From the voltammetric measurement the band edge parameters viz. valance band and conduction band edge were estimated vs. NHE and local vacuum respectively. Along with band edge parameters the crystals structure parameters like lattice constants and d-spacing calculated from X-ray diffraction pattern also presented in Table 1.

## Figures and Tables

**Table 1:** The optical band gap, electrochemical band gap with band edge positions vs. NHE. local vacuum and lattice constants as a function of composition for CZTS*_x_*Se_1−*x*_ alloy nanocrystals.

**Sr. no**	**Nanocrystal composition**	**Optical band gap**$\left(\varepsilon_{gap}^{op}\right)$eV	**Electrochemical band gap**$\left(\varepsilon_{gap}^{ec}\right)$	***E**_**VB**_***Vs. vacuum (V)**	***E**_**CB**_***Vs. vacuum (V)**	***E**_**ox**_***Vs. NHE (eV)**	***E**_**red**_***Vs. NHE (eV)**	**Lattice constant ‘*a*’ (Å)**	**Lattice constant ‘c’ (Å)**	**d**_**112**_**spacing (Å)**
1	CZTS*_x_*Se_1−*x*_(*x*=1)	1.50	1.55	−5.49	−3.94	0.99	−0.56	5.4300	10.6900	3.1304
2	CZTS*_x_*Se_1__−*x*_(*x*=0.74)	1.40	1.48	−5.33	−3.85	0.85	−0.65	5.5200	10.9500	3.1841
3	CZTS*_x_*Se_1−*x*_(*x*=0.42)	1.32	1.39	−5.44	−4.05	0.96	−0.45	5.5800	11.1200	3.2281
4	CZTS*_x_*Se_1−*x*_(*x*=0.17)	1.24	1.16	−5.46	−4.3	0.94	−0.20	5.6100	11.2500	3.2594
5	CZTS*_x_*Se_1−*x*_(*x*=0)	1.16	1.08	−5.55	−4.47	1.05	−0.03	5.6890	11.3400	3.2818

## References

[1] Jadhav Y.A., Thakur P.R., Haram S.K. (2016). Voltammetry investigation on copper zinc tin sulphide/selenide (CZTS*_x_*Se_1−*x*_) nanocrystals: estimation of composition dependent band edge parameters. Sol. Energy Mater. Sol. Cells.

[2] Guo Q., Hillhouse H.W., Agrawal R. (2009). Synthesis of Cu_2_ZnSnS_4_ nanocrystal ink and its use for solar cells. J. Am. Chem. Soc..

[3] Riha S.C., Parkinson B. a, Prieto A.L. (2011). Compositionally tunable Cu_2_ZnSn(S_1−_*_x_*Se*_x_*)_4_ nanocrystals: probing the effect of Se-inclusion in mixed chalcogenide thin films. J. Am. Chem. Soc..

[4] Mourdikoudis S., Liz-Marzan L.M. (2013). Oleylamine in nanoparticle synthesis. Chem. Mater..

[5] Bradley D.C., Gitlitz M.H. (1968). Metal–nitrogen infrared stretching frequencies in Dialkylamido-transition metal compounds. Nature.

